# Uncovering Genetic Diversity and Adaptive Candidate Genes in the Mugalzhar Horse Breed Using Whole-Genome Sequencing Data

**DOI:** 10.3390/ani15182667

**Published:** 2025-09-11

**Authors:** Shinara N. Kassymbekova, Zhanat Z. Bimenova, Kairat Z. Iskhan, Przemyslaw Sobiech, Jan P. Jastrzebski, Pawel Brym, Wiktor Babis, Assem S. Kalykova, Zhassulan M. Otebayev, Dinara I. Kabylbekova, Hasan Baneh, Michael N. Romanov

**Affiliations:** 1 Department of Clinical Disciplines, Faculty of Veterinary and Zooengineering, Kazakh National Agrarian Research University, Almaty 050010, Kazakhstan; shinarakassymbekova@gmail.com (S.N.K.); zhanat.bimenova@kaznaru.edu.kz (Z.Z.B.); a.kalykova@gmail.com (A.S.K.); dinakabylbekova3@gmail.com (D.I.K.); 2 Department of Animal Biology Named after N.U. Bazanova, Faculty of Veterinary and Zooengineering, Kazakh National Agrarian Research University, Almaty 050010, Kazakhstan; model.univer@gmail.com (K.Z.I.); otebayev.zhassulan@kaznaru.edu.kz (Z.M.O.); 3 Department and Clinic of Internal Diseases, Faculty of Veterinary Medicine, University of Warmia and Mazury in Olsztyn, 10-719 Olsztyn, Poland; psobiech@uwm.edu.pl; 4 Department of Plant Physiology Genetics and Biotechnology, Faculty of Biology and Biotechnology, University of Warmia and Mazury in Olsztyn, 10-719 Olsztyn, Poland; 5 Department of Animal Genetics, Faculty of Animal Bioengineering, University of Warmia and Mazury in Olsztyn, 10-719 Olsztyn, Poland; pawbrym@uwm.edu.pl; 6 Ecology and Genetics, Faculty of Science, University of Oulu, 90520 Oulu, Finland; wiktorbabis@gmail.com; 7 Department of Fundamental Medicine, al-Farabi Kazakh National University, Almaty 050040, Kazakhstan; 8 Project Center for Agro Technologies, Skolkovo Institute of Science and Technology (Skoltech), Moscow 121205, Russia; hasanbaneh@gmail.com; 9 Animal Science Research Department, Kurdistan Agricultural and Natural Resources Research and Education Center, Agricultural Research, Education and Extension Organization (AREEO), Sanandaj 6616936311, Iran; 10 School of Natural Sciences, University of Kent, Canterbury CT2 7NJ, UK; 11 Animal Genomics and Bioresource Research Unit (AGB Research Unit), Faculty of Science, Kasetsart University, Chatuchak, Bangkok 10900, Thailand; 12 L. K. Ernst Federal Research Center for Animal Husbandry, Dubrovitsy, Podolsk 142132, Russia

**Keywords:** Mugalzhar horse, whole-genome sequencing (WGS), single nucleotide polymorphisms (SNPs), inbreeding, adaptation

## Abstract

Mugalzhar horses are a relatively young native breed of Kazakhstan, prized for meat and milk production and adaptation. This study was conducted to investigate genetic diversity and pinpoint genomic regions associated with selection signatures in this breed using whole-genome sequence data. Variant calling yielded a total of 21,722,393 high-quality variants, including 19,495,163 SNPs and 2,227,230 indels. Most variants were located in introns and intergenic regions, while only 1.94% were exonic. Estimates of genetic diversity were moderate, with expected and observed heterozygosity and nucleotide diversity of 0.2325, 0.2402, and 0.0021, respectively. We identified nine adaptive candidate genes (*SCAPER*, *FHAD1*, *MMP15*, *ADGRE1*, *CMKLR1*, *MRPL15*, *ZNF667*, *CCDC66*, and *LOC100055310*), harboring high-impact exonic variants in the homozygote state for an alternative allele. No deleterious segregating variants associated with Mendelian traits were found in this population, while seven variants linked to coat color and gaitedness were detected in a low frequency heterozygous state. Our findings suggest that there are certain genomic regions subjected to ancient selection footprints during the ancestor breed formation and adaptation. The outcome of this study serves as a foundation for future genomic-driven strategies, a broader utilization of this breed, and a reference for genomic studies on other horse breeds.

## Simple Summary

Mugalzhar horses are a native breed of Kazakhstan valued for their ability to produce milk and meat and adapt to harsh environments. This study explored the genetic diversity of these horses and identified regions of their DNA affected by natural selection using advanced genome analysis techniques. Using more than 21 million genetic variants, we found that most of them occurred in non-coding regions of the genome, with only a small fraction affecting certain genes directly as candidates for adaptation to a harsh climate. Despite the presence of rare genetic markers associated with traits like coat color and gait, no harmful segregating genetic mutations linked to diseases with Mendelian inheritance were identified. These results suggest that Mugalzhar horses have maintained a moderate genetic diversity, exhibiting traces of historical selection and no signs of inbreeding. This study provides useful insights into the genetic makeup of this breed, which can help to preserve and improve it in breeding programs.

## 1. Introduction

Recent genomic studies [1,2,3] suggested that the horse was domesticated around 5500 years ago, significantly later than the domestication of most other livestock species. Despite this, horses (*Equus caballus*) have considerably influenced human civilization [4,5,6], having made a significant contribution to cultural exchange between societies, economic development, and supporting the farmers in agricultural activities [7,8]. The recent discovery of ancient equine signature tracks in the horse genome has led scientists to propose Kazakhstan as a candidate site for the initial domestication of horses [9,10]. Horse breeding has been considered an important part of the traditional livestock production sector in Kazakhstan for centuries, which has led to generations of herding knowledge. According to Kosharov et al. [11], steppe horses from Kazakhstan were widely distributed as early as the fifth century B.C. The country’s vast territory of natural pastures (185 million hectares), the constant demand for horse products, and a high cultural affinity for traditional equestrian sports have provided a strong potential for the development of horse breeding [12,13]. As of 1 January 1980, there were 312,447 Kazakh horses, including 63,329 purebreds, which are kept on pastures all year round.

**Figure 1:**
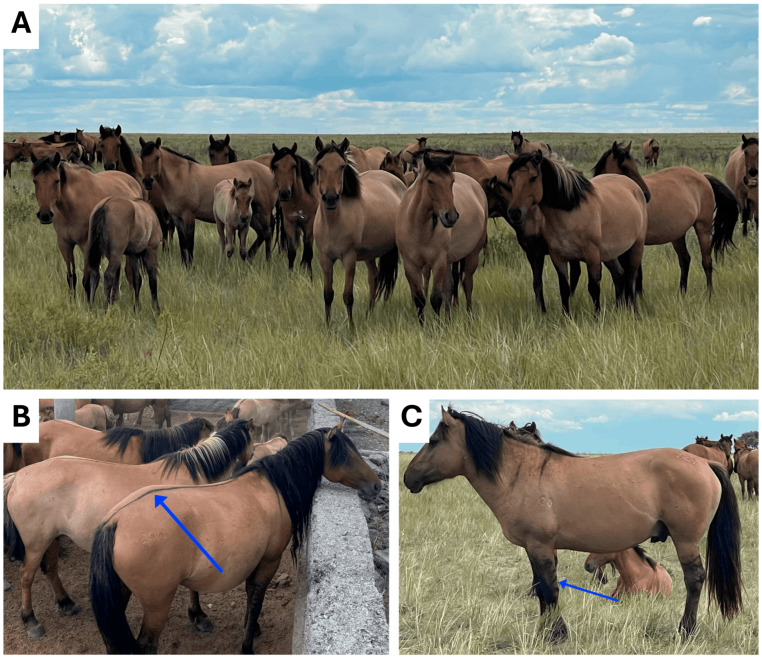
(**A**) The Mugalzhar horse breed (Photo by K. Iskhan); (**B**) The Mugalzhar horse breed with «zebra pattern» stripes on the legs; (**C**) and characteristic «dorsal stripe» (Photo by T. Assanbayev).

A native Mugalzhar breed (Figure 1A) [14,15], named after the area (i.e., Mugalzhar District, including the village of Mugalzhar) where it was bred, had been developed through a selective breeding program applied to Jabe-type Kazakh horses, between 1969 and 1998, in order to enhance the meat and milk production [16]. In 1998, this breed was officially recognized as a new breed by the Ministry of Agriculture of Kazakhstan (Order No. 156, dated 30 December 1998) [17,18,19,20,21]. By 2020, the breed population embraced 16,290 horses, including 7026 mares [12].

Mugalzhar horses, mainly of dun or bay coat colors without spots, exhibit a compact physique with a height at the withers not exceeding 145 cm. The Mugalzhar horse populations and their ancestral breed Jabe frequently exhibit a “zebra pattern” leg stripes (Figure 1B) and a “dorsal stripe” (Figure 1C). These phenotypic traits likely reflect the preservation of ancient equine genetic lineages [22]. This breed is distinguished by its potential to produce high-quality horsemeat and by substantial milk yield, even under a year-round pasture-grazing production system [23,24,25]. Iskhan et al. [16] claimed that this is the world’s first dual-purpose (meat and dairy) breed developed from a non-specialized local horse breed, which provides high-quality meat and kumis during year-long grassland farming [23]. It has a faster growth rate and, accordingly, a better meat productivity, with body weight being 100–120 kg more for stallions and 80–100 kg more for mares compared to its Jabe-type ancestor [26]. In particular, stallions typically weigh between 493.5 and 538.4 kg, while mares range from 452.7 to 469.3 kg [16]. Owing to these distinctive characteristics, the Mugalzhar horse is considered a valuable breed in Kazakhstan [27]. However, this breed is distributed across the whole country; they are mainly reared in the Karagandy region of central Kazakhstan, the Aktobe region of western Kazakhstan, and the Kyzylorda region of southwest Kazakhstan [28].

According to Iskhan et al. [16], there were three main intra-breed types of the Mugalzhar breed, i.e., Emba, Kulandy, and Kozhamberdy (till 2009 known as Saryarqa), as well as six lines and 55 families in 2019. The six lines listed by Iskhan et al. [16] were Maupas and Mesker (of Kozhamberdy type), and Patok, Zaliv, Aral, and Kulan (of Kulandy type), named after the respective outstanding stallion. In 2024, Kabylbekova et al. [12] stated that the modern breed structure consists of four intra-breed types (Emba, Kulandy, Saryarqa, and, since 2009, Kozhamberdy), six lines, and 55 families. In 2024, Iskhan et al. [25] also described a new Irtysh stud farm type of the Mugalzhar breed [29,30]. Within the Kozhamberdy intra-breed type of the Mugaldzhar breed, there are structurally two established stud farm subtypes, i.e., Saryarqa and Kaindy. Orazymbetova et al. [31] mentioned five Kozhamberdy lines. Exploring three of them, i.e., Maupas, Mesker, and Meiman, Orazymbetova et al. [31] showed that they are genetically divergent. These subtypes and lines are part of the selective breeding work aimed at improving the breed and developing its specialized herds. A more detailed account pertinent to the Mugalzhar breed history, its breeding, and production features is presented in Supplementary Information.

Adequate genetic diversity within the population is essential for a successful selective and sustainable breeding program and genetic progress in Mugalzhar horses. It also helps with monitoring inbreeding level in this population and reducing inbreeding depression, as a potential threat to production and climate resilience. Additionally, identifying the candidate genes linked to economic traits enables integrating genomic information into breeding decisions and consequently more efficient and breed-specific selection strategies. The genetic studies of Kazakh horse breeds over the past decade have mainly focused on assessing genetic diversity and identification of candidate genes associated with economically important traits using a limited set of microsatellite markers [28,31,32]. Investigations using single-nucleotide polymorphism (SNP) array genotypes have unraveled significant genetic differences between native Kazakh and other exotic horse breeds [33,34]. Although SNP chips are informative, with genetic markers dispersed over the whole genome [35,36,37,38,39], they cover a part of genomic variation and, in particular, are not able to detect structural variants.

Whole-genome sequencing (WGS) has revolutionized the investigation of genetic architecture in livestock and wild species [40,41,42,43], guiding their comprehensive assessments of genetic diversity [44,45,46], inbreeding [47,48,49], and population structure [50,51,52]. This high-resolution approach provides insights into evolutionary history and detection of the selection footprints and breed-specific variants [53,54,55]. Previous equine genomic studies have used WGS to assess the genetic diversity, evaluate the inbreeding level, and identify genomic regions underlying economically important traits in horses [56,57]. A recent investigation [58] of runs of homozygosity (ROH) using WGS revealed population-specific inbreeding patterns in different horse breeds. These findings highlighted the importance of understanding inbreeding dynamics, particularly in indigenous breeds, to guide conservation and breeding strategies. Despite the economic and cultural importance of the Mugalzhar horse breed in Kazakhstan, the genomic characteristics of this breed remain largely unexplored.

Previously, we suggested that the Mugalzhar DNA samples obtained for the microsatellite research [33] would enable performing their WGS to seek breed-specific SNP variants. In the present study, we presented the first whole-genomic sequence analysis of the native Mugalzhar horse breed, aiming to establish fundamental genomic characteristics, including the assessment of genetic diversity and level of inbreeding, and the identification of genome regions under selection that will lay a foundation for breeding strategies in this unique Kazakhstani breed.

## 2. Materials and Methods

### 2.1. Animals and Sample Collection and DNA Extraction

In this investigation, blood samples from 20 healthy Mugalzhar horses were collected at the ORDA breeding farmer enterprise headed by Amandyk Zhumagalyuly, in the vicinity of Khromtau (Shalkar District, Aktobe Region, western Kazakhstan; 50°15′04″ N, 58°26′24″ E; 429 m elevation above sea level). All 20 individuals were previously sampled for the microsatellite-based investigation [32]. They represented the local Aktobe population and belonged to the Meiman line of the Kozhamberdy intra-breed type that represents a selective breeding achievement developed within this intra-breed type. A more detailed account of the sampling site and sampled animals is provided in Supplementary Information.

Horse gender, birth year, and coat color of the collected samples were recorded and are provided in Supplementary Table S1 and in more detail in Supplementary Information. Genomic DNA isolation was performed using the phenol–chloroform procedure following Maniatis et al. [59]. According to DNA concentration and quality metrics, assessed by A260/A280 and A260/A230 ratios, the extracted genomic DNA for all samples, shown in Supplementary Table S2, was suitable for downstream genomic analyses.

### 2.2. WGS, Reads Preprocessing and Mapping

This high-molecular-weight DNA was then sequenced using Illumina’s (San Diego, CA, USA) NovaSeq 6000 sequencing technology that provided 150-bp paired ends. After quality controls of the raw reads, Trimmomatic software (version 0.39; [60]) was used to pre-process the reads and remove the adapter sequences (the first 10 nucleotides), low-quality, and short reads. We compared two sequence quality thresholds (Q20 and Q30), and applied Q20, which provides a suitable and acceptable efficient alignment, with a low rate of sequence removal, resulting, on average, in 380,480,205 reads per sample and ranging from 216,143,288 to 562,962,552 reads per horse. The average read sequencing quality Phred scores >35 and >20 were 94.84% and 99.86%, respectively.

High-quality sequence alignment was achieved, with trimmed reads mapping uniquely to the horse reference genome assembly EquCab3.0 (with annotation GCF_002863925.1 [61]), at an average rate of 99.79%, using Burrows–Wheeler Aligner (BWA) software (version 0.7.17-r1188; [62]) with default parameters. Herewith, average allele frequency, sequencing depth, and BaseQRankSum statistics of the identified variants were determined. The aligned mapping files (SAM files) were sorted and converted to binary format (BAM files) and merged to obtain individual files for each horse separately using SAMtools (version 1.6; [63]). The merged and sorted BAM files were then indexed using SAMtools for further analysis.

### 2.3. Variants Calling and Annotation

The Genome Analysis Toolkit GATK4-4.0.5.1-0 (GATK) pipeline using haplotypecaller [64] was used for variant calling. To reduce the risk of error and facilitate further analysis, the following two pipelines were used to generate the result files of all variants: (1) an analysis combining all BAM files and calling variants with all trials included; and (2) an independent analysis individually for each horse, combining then all variant files into a single file. Variant Effect Predictor (VEP) tools [65] were used to annotate the functional, location, and classification of identified variants. The variants identified in this study, along with their predicted effects by functional annotation classes, were visualized using Circos (version 0.69-9; [66]).

In order to identify Mugalzhar breed-specific variants, the following two criteria were applied for filtering those variants: (1) based on the variants with at least 10-nucleotide position coverage depth that were sequenced in each sample at least 10 times; and (2) variants with fixed (AF = 1) alternative alleles. In other words, we selected only those variants that were at homozygous state for alternative alleles in all 20 studied samples. The variants were functionally annotated using the Variant Effect Predictor (VEP) tool [65] and Ensembl ([67]; release 114, published on 7 May 2025 [68]) annotation of the horse genome assembly EquCab3.0 [61]. Only those genes harboring ≥5 exon variants with “high” or “moderate” impact (according to the VEP analysis) were retained and considered as prioritized candidate genes (PCGs).

### 2.4. Genetic Diversity Analysis and Population Structure

The genome-wide observed (*H_o_*) and expected (*H_e_*) heterozygosity values were computed using VCFtools (version 0.1.16; [69]) for processing the Variant Call Format (VCF) data. We also estimated nucleotide diversity (π) that reflects, on average, the quantity of nucleotide variations per site between two DNA sequences selected at random from the population under study [70]. The nucleotide diversity was estimated over the entire autosomal genome using a sliding window approach implemented in VCFtools (version 0.1.16; [69]).

In order to provide a more reliable and comprehensive overview of inbreeding in this population, three methods (-ibc function) implemented in the GCTA program (version 1.94.4; [71]) were applied to estimate the genomic inbreeding, as follows:

1. *F*_GRM_, the inbreeding coefficient driven from the genomic relationship matrix (GRM) and calculated as the deviation of the diagonal elements from unity:
  (1) $$GRM=\frac{ZZ^{\prime }}{2\sum p_{i}(1\;-\;p_{i})}\;F_{{GRM}_{i}}={GRM}_{ii}-1,$$
  where *Z* is the standardized and centralized genotype file, *p* is the minor allele frequency, $F_{{GRM}_{i}}$ is the inbreeding coefficient for *i*th sample, and ${GRM}_{ii}$ is the *i*th diagonal element of GRM corresponding to *i*th sample.
2. *F*_HOM_, the Wright’s inbreeding coefficient based on the proportion of the loci with higher observed homozygosity than expected homozygosity:
  (2) $$F_{HOM}=\frac{N_{O\left(Hom\right)}-N_{E\left(Hom\right)}}{N_{Non\_Miss}-N_{E\left(Hom\right)}\prime }$$
  where $N_{O\left(Hom\right)}$, $N_{E\left(Hom\right)}$ and $N_{Non\_Miss}$ are numbers of observed homozygous, expected homozygous, and non-missing loci, respectively.
3. *F*_UNI_, the Wright’s inbreeding coefficient based on the correlation between alleles in uniting gametes:
  (3) $$F_{UNI}=\frac{1}{n}\sum_{i=1}^{n}\left(\frac{x_{i}^{2}-\left(1+2p_{i}\right)x_{i}+2p_{i}^{2}}{2p_{i}(1-p_{i})}\right),$$
  where $x_{i}$ is the number of copies of the reference allele for the *i*th SNP, *n* is the number of versions of the reference allele, and $p_{i}$ is the minor allele frequency for the *i*th SNP.

In order to investigate the population stratification of the studied samples, principal component analysis (PCA) was implemented based on GRM. Before constructing GRM, the dataset was filtered for biallelic SNPs with MAF > 0.05, resulting in 13,536,151 SNPs. GRM was constructed using GCTA (version 1.94.4; [71]). PCA plots were produced using Microsoft Excel and *ggplot2* [72] in the R environment [73,74].

### 2.5. Segregating Variants from Online Mendelian Inheritance in Animals

To investigate variants associated with Mendelian traits in horses, we obtained the genomic position and the corresponding phenotypes of 116 trait-associated variants from the Online Mendelian Inheritance in Animals (OMIA) database [75]. Segregating variants was selected based on the number of heterozygote genotypes in the samples.

## 3. Results

### 3.1. WGS, Reads Preprocessing, Mapping, Variant Calling, and Annotation

High-quality extracted genomic DNA with enough DNA concentration led to high-quality sequencing and consequently efficient alignment of sequence reads (99.79%) over the reference genome, as summarized in Table 1. WGS information in more detail is available in the Supplementary Table S3.

**Figure 2:**
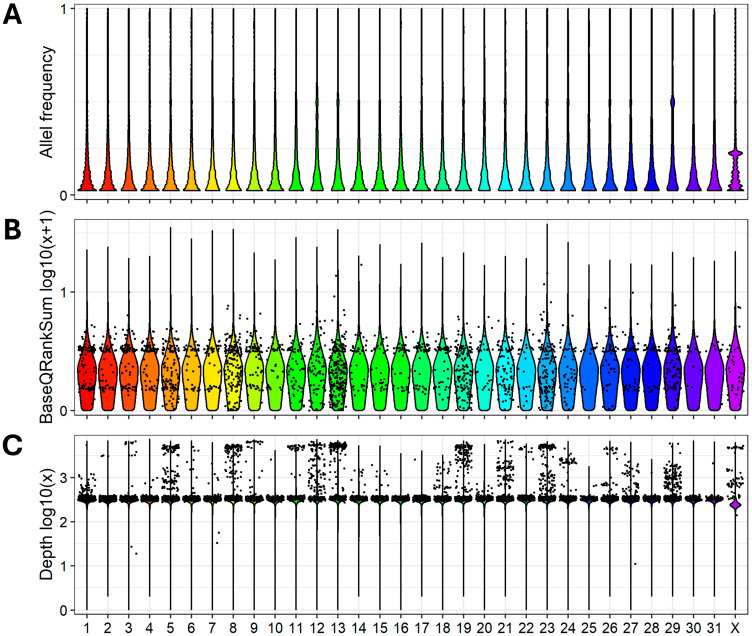
Violin plots for all identified variants in each of 32 horse chromosomes: (**A**) density of allele frequency, (**B**) density of BaseQRankSum statistics, and (**C**) shows density of sequencing depth. Data are pooled from all 20 Mugalzhar horses.

Average allele frequency, BaseQRankSum statistics, and sequencing depth for the identified variants were visualized within each chromosome as shown in Figure 2.

The variant calling analysis resulted in sites of 21,722,393 variants, including 19,495,163 SNPs and 2,227,230 indels. Among these, 20,354,948 variants (93.7%), including 18,364,296 SNPs and 1,990,652 indel variants, were located on 32 chromosomes, both autosomes and sex chromosomes. The distribution of the variants in different classes based on the number of alleles is shown in Supplementary Table S4. As expected, most of the variants (*n* = 19,958,242; 91.88%) were biallelic loci, including 18,245,338 SNPs (91.49%) and 1,712,904 indels (8.51%). The distribution of these variants on 32 chromosomes is given in Table 2. Around 96% of the biallelic variants (19,123,873 variants, 17,495,490 SNPs, and 1,628,383 indels) were located on autosomes.

Functional annotation of the identified biallelic variants is summarized in Table 3. The results showed that the majority of both SNPs and indels are intergenic variants (75.34% and 72.62%, respectively) or located in intronic regions (18.41% and 20.55%, respectively). There were only 353,056 biallelic SNPs located in exon regions, which constituted 1.94% of the total identified biallelic SNPs in this breed. Of those SNPs, 62,111, 42,319, and 7112 variants were respectively missense, synonymous, and high-impact SNPs (splice donor, splice acceptor, stop gained, stop lost, and start lost variants). Among the missense variants, 1945 SNPs were identified as deleterious to protein function according to SIFT score criteria (SIFT < 0.05). Among the 6323 protein-coding indels identified, 5048 are high-impact variants, with frameshift variants being the most frequent class, comprising 4444 variants.

### 3.2. Genetic Diversity Analysis and Population Stratification

In terms of the examined population heterozygosity, the average genome-wide estimated *H_o_* and *H_e_* values were 0.2402 and 0.2325, respectively. In addition, the average genome-wide nucleotide diversity (π) within the Mugalzhar horse breed was estimated to be 0.0021 bp^−1^.

The descriptive metrics for the estimated inbreeding coefficients for the studied samples are provided in Table 4. The average inbreeding coefficients in all three methods were negative and were −0.038, −0.033, and −0.033 for *F*_GRM_, *F*_HOM,_ and *F*_UNI_, respectively.

**Figure 3:**
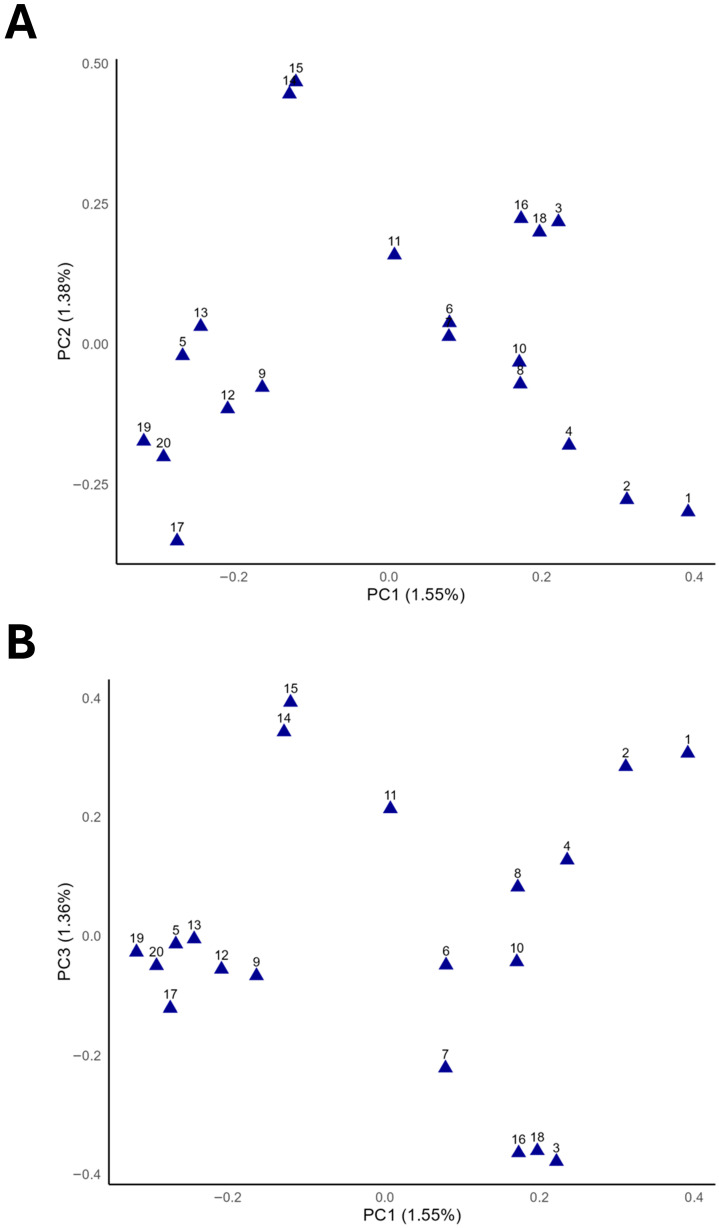
Principal component analysis (PCA) of the population structure and genetic relationships between 20 Mugalzhar individuals: (**A**) distribution of the individuals in the projections of two coordinates, i.e., the first (PC1; X-axis) and second (PC2; Y-axis) principal components, showing percentage of total genetic variation that can be explained by each of the two PCs (indicated within the parentheses); (**B**) same in the dimensions of two coordinates, i.e., PC1 (X-axis) and the third principal component (PC3; Y-axis).

The PCA results and the respective plots for 20 individuals are presented in Figure 3 and show the sampled population structure. In this figure, principal component 1 (PC1) is plotted against second (PC2) and third (PC3) components (Figure 3A and Figure 3B, respectively). The PCA plot reveals the genetic diversity among 20 Mugalzhar horse samples, with PC1 (1.55%) and PC2 (1.38%) explaining the total variation. A wide range of principal components obtained for the samples indicates a high level of diversity in the population. However, there are some small subgroups of two or more individuals, indicating possible population stratifications. The scatterplot PC1 by PC2 is basically similar to PC1 by PC3, pointing out that, most likely, the first three PCs do not contrast any variation in the genome of the Mugalzhar breed and correspond to specific features of this breed.

### 3.3. Adaptation Footprints and Candidate Genes

**Figure 4:**
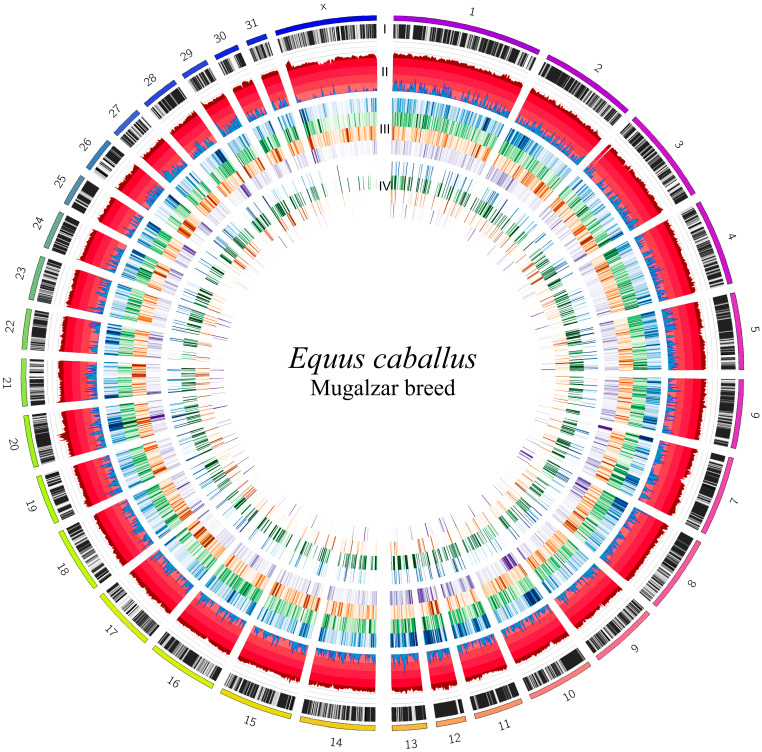
Circos (version 0.69-9; [66]) plot showing 32 chromosomes of *Equus caballus*. Chromosomes are divided into bins of 1,000,000 nucleotides. Description from the outermost to the innermost circle: I, rainbow scale colored bars are the chromosomes and black/white barcodes means gene concentration in the reference genome (EquCab3.0; [61]); II, concentration of variants detected in our data (red) and concentration of variants passing the allele frequency and sequencing depth filtering (blue); III, frequency of detected effects of variants (using Variant Effect Predictor (VEP) tool [65]) divided into four groups: intron (blue), intergenic (green), up- or downstream gene (orange), and other (purple); IV, concentration of detected consequences of variants obtained after the filtration step and divided into four groups: intron (blue), intergenic (green), up- or downstream gene (orange), and other (purple). Darker colors on heatmap indicate higher concentrations.

Since the variants with high allele frequency are very important for breed-specific analysis, we kept only the variants whose alternative allele is fixed in this population. Therefore, 139,163 variants with allele frequency for the alternative allele equal to unity were identified. It means that the reference allele of those variants was not observed in all 20 samples analyzed. Therefore, these variants were kept for further analysis. In order to improve the reliability of the results and reduce the risk of random error, we applied a depth-of-coverage filter, retaining only variants supported by at least 10 sequencing reads (≥10X) in each sample, which resulted in 15,027 high-confidence variants. The selected threshold was because of the fact that the lower coverage is associated with reduced genotyping accuracy and higher false-positive rates. A graphical summary of these analyses is shown in Figure 4.

Among these variants, we retained only 9318 variants that are located on autosomal chromosomes. In total, 73 exon variants located on nine protein-coding genes passed the criteria for PCGs. These PCGs harboring high-impact homozygous exon variants that are fixed in Mugalzhar horses are listed in Table 5.

These genes were located on several autosomal chromosomes, including ECA1 (*SCAPER*), ECA2 (*FHAD1*), ECA3 (*MMP15*), ECA7 (*ADGRE1*), ECA8 (*CMKLR1*), ECA9 (*MRPL15*), ECA10 (*ZNF667*), ECA16 (*CCDC66*), and ECA23 (*LOC100055310*). PCGs have a high number of orthologous genes, in a range of 30 (*LOC100055310*) to 344 (*CMKLR1*). Among the detected genes, *MRPL15* conformed to a remarkably high number of homozygous exon variants for the alternative allele (*n* = 15) with high impact.

### 3.4. OMIA Variants Segregating Analysis

To investigate variants associated with Mendelian traits in horses, we analyzed 116 previously reported variants across various breeds. The results revealed that seven variants in four genes (*MC1R*, *KIT*, *MITF*, and *DMRT3*) are segregating in the studied horse population. The detailed description of these variants, their associated genes, and phenotypes is presented in Table 6. A missense mutation (ECA3:g.36979560C > T) of the *MC1R* gene associated with coat color phenotype (OMIA 001199-9796 [75]) was found in a heterozygous state in four horses, indicating moderate segregation of this allele in the population. Three missense mutations (ECA3:g.79538738C > T, ECA3:g.79548220T > C, and ECA3:g.79566881T > C) of the *KIT* gene were also observed in the heterozygous state in only one horse. These variants are known to influence phenotypes such as white spotting and dominant white coat color (OMIA 000209-9796 [75]). In the *MITF* gene, one structural variant (ECA16:g.21555811delinsAAAT) and a regulatory variant (ECA16:g.21608936C > T) are segregating in this population, which are associated with the splashed white phenotype (OMIA 000214-9796 [75]). A stop-gain SNP (ECA23:g.22391254C > A) of the *DMRT3* gene was identified in a heterozygote state in one horse, which is associated with gaitedness, a distinctive pattern of movement in horses (OMIA 001715-9796 [75]).

## 4. Discussion

### 4.1. WGS Outcome and Variant Characterization in the Mugalzhar Breed

Understanding the genetic diversity within livestock breeds is crucial as it influences the success of effective selective breeding programs [76,77,78,79,80] and the preservation of genetic potential for adaptive traits (e.g., survival in specific environmental conditions), helping prevent excessive rate of inbreeding [81,82,83,84,85]. Whole-genome sequences are a promising tool for investigating the evolutionary history, selection signatures, and breed-specific genomic profile, assessment of genetic diversity, and identifying genomic regions underlying economically important traits in livestock species [53,54,55,56,57]. In this study, we identified 21,722,393 variants, including 2,227,230 indels and 19,495,163 SNPs among 20 individuals of the Mugalzhar horse, representing a significant contribution to the current resources available for equine research. Although high-quality sequencing data provided valuable insights into the genomic structure of the breed, the limited sample size (n = 20) may reduce the power to detect rare genetic variants.

The total number of autosomal variants (19,502,882) identified in this breed was higher than those reported for North American [86] and Japanese Thoroughbred horses [87]. These differences could be due to the evolutionary history of the breeds, sampling criteria, sequencing technology, and applied bioinformatic procedure. However, similar findings have been reported by Al Abri et al. [88]. The proportion of bi-allelic indel variants (86.05%) was lower compared to that of bi-allelic SNPs (99.35%). It has been reported that indels are underlying some genetic disorders in horses, like lavender foal syndrome [89] and severe combined immunodeficiency [90]. The number of exonic SNPs (353,056; ~1.94%) was close to the value reported by Al Abri et al. [88]. The average genome-wide SNP density for autosomes was 1/130 nucleotides, ranging from 1/87 (ECA 12) to 1/147 (ECA 13). This was approximately 10 times higher than the density of indels, which had an average of 1/1205 and ranged from 1/873 (ECA 20) to 1/1328 (ECA 9).

### 4.2. Genomic Diversity, Inbreeding, and Population Stratification

The average of genome-wide nucleotide diversity, a metric providing valuable insight into the divergence, demographic history, and genetic diversity of populations [70], was estimated to be 0.0021 bp^−1^. This value indicates that, on average, there were ~2 nucleotide differences per 1000 base pairs between two randomly selected sequences in this horse population, corresponding to a total of 5,250,000–5,670,000 nucleotides across the 2.5–2.7 Gb horse genome size [91]. The estimates of these genomic diversity metrics indicate a moderate level of genetic variability in the Mugalzhar horse population. Our estimate is higher than the average diversity (0.0017) in the autosomal chromosomes reported for six other horse breeds (American Miniature, Percheron, Arabian, Mangalarga Marchador, Native Mongolian Chakouyi, and Tennessee Walking) by Al Abri et al. [88].

A higher average genome-wide *H_o_* value (0.2402) compared to *H_e_* (0.2325) indicates the preservation of genetic diversity and a relatively low level of inbreeding in this population from the Aktobe Region. Using SNP microarrays, Pozharskiy et al. [33] reported a high diversity level for 584 Mugalzhar horses, with *H_o_* and *H_e_* estimates being 0.345 and 0.340, respectively, which exceed our findings for a smaller Mugalzhar sample size. Genomic inbreeding coefficients reflect the actual level of homozygosity in the genome, which is not affected by the availability, depth, accuracy, and completeness of the pedigree information [92]. In this study, we applied three different methods to estimate inbreeding, which allows for comparison between estimates, minimizes method-specific biases, and provides more robust results. The negative average genomic inbreeding coefficients obtained in this study using three methods (i.e., *F*_GRM_, −0.038; *F*_HOM_, −0.033; and *F*_UNI_, −0.033) suggest that the Aktobe population of Mugalzhar horses exhibits a relatively high level of variability, supporting adequate genetic progress in a selection program. PC 1-3 explained a relatively low proportion of the variation in the studied population, which could be due to the high number of original variables (e.g., millions of WGS variants) and the genetic homogeneity of the breed. The distribution of individuals on PCA plots showed a wide scatter, while the similarity in PCA scatterplots, on the other hand, suggests that the observed population structure within the studied samples might be due to a relationship pattern among the animals over recent generations (e.g., due to the development of the Meiman line).

The moderate level of genetic variability could be due to the random mating, a relatively high ratio of breeding males to females, and early animal culling in a round-year grazing and meat production-oriented system. The genetic diversity in this breed, as indicated by several metrics outlined above, suggests that the horse population can be utilized to provide genetic diversity for other breeds and has the potential to further boost productivity. The previously published findings using 17 microsatellite markers in the same population [32] demonstrated higher *H_o_* and lower inbreeding values, confirming the results of the current study. Additionally, in a wider microsatellite examination of Kazakh horses, including the Mugalzhar breed, Orazymbetova et al. [31] identified minimal genetic differentiation (0.05%), suggesting significant admixture and an ongoing lineage sorting process, which is consistent with our results.

### 4.3. PCGs

There were 9318 autosomal variants with a homozygous state for the alternative allele in all 20 studied samples. Among them, only the exonic variants with high or moderate impact, according to VEP, were retained. The genes harboring at least five variants were considered as potentially candidate genes (PCGs) associated with fitness/adaptation in this breed. The identified genes represent strong candidates for further investigation; however, functional and experimental validation is recommended to confirm their biological impacts on adaptation and economically important traits in Mugalzhar horses. The functions and potential relevance to horse biology, and particularly for the Mugalzhar breed’s adaptability, of these PCGs are outlined below.

*SCAPER* (S-phase cyclin A-associated protein in the endoplasmic reticulum) is mainly involved in nucleic acid binding, which has been reported to be associated with nonsyndromic intellectual disability [93] and retinal disease [94] in humans, male sterility and reduced female fertility in mice [95], sperm motility in Holstein cattle [96], adaptation in the cattle [97], growth and nervous system in goats [98], and as a potential deleterious gene selected in Tibetan pigs [99]. *FHAD1* (forkhead associated phosphopeptide binding domain 1) encodes a protein that acts as a regulator of sperm motility and spermatocyte meiosis. This gene has been reported to be located in the genomic regions under potential selection in adaptation in the Tunisian Black Thibar sheep [100], highly expressed in remyelinating lesions [101], and associated with body weight and size in the Jabe horse breed, which is the ancestor of Mugalzhar horses [34]. The Mugalzhar horses are reared in a year-round grazing system, where the reproductive efficiency is an important factor for this production system. Meanwhile, a higher growth rate helps the animals, especially the young horses, become stronger and more resilient to harsh climates when food availability is limited. It appears that the *SCAPER* and *FHAD1* genes may contribute to the Mugalzhar breed’s adaptability by enhancing the summer growth rates, thereby improving physiological robustness for survival during harsh winters.

*MMP15* (matrix metallopeptidase 15) encodes a member of the peptidase M10 family that plays a role in the breakdown of extracellular matrix during both disease processes and normal physiological processes. It was reported [102] that this gene is located in the positive selection signature in the live-bearing fish *Heterandria formosa*, which contributes to endometrial tissue remodeling and placental labyrinth formation. Dierks [103], using GWAS in Hanoverian warmblood horses, reported a QTL associated with osteochondrosis and osteochondrosis dissecans, which is the genomic region with *MMP15* homology in the human genome. This gene is not well-studied, even in the other livestock species, and its biological role in improving the adaptability of this horse breed requires further investigations.

*ADGRE1* (adhesion G protein-coupled receptor E1) contains a domain similar to seven seven-transmembrane G protein-coupled receptor and plays a role in cell adhesion and interactions between cells, particularly immune system cells. This gene plays an important role in the resistance and resilience of the Mugalzhar horses, as a native breed, to environment-specific pathogens. Its function has been reported to be associated with defense against infections through the development of antigen-specific CD8+ regulatory T cells [104,105,106]. It has also been reported that it is involved in both innate and adaptive immune responses, and was identified as a target of positive selection against tropical parasites in African dogs [40].

*CMKLR1* (chemerin chemokine-like receptor 1) regulates negatively NF-kappaB transcription factor activity, which positively regulates the macrophage chemotaxis, and regulation of calcium-mediated signaling, adipogenesis, and adipocyte metabolism. Dander et al. [107] suggested that the CMKLR1/chemerin axis controls intestinal graft-versus-host disease. While de Camargo et al. [108] found that this gene is associated with protein percentage in dairy buffaloes. Evidence suggests that the RARRES2-CMKLR1 axis may be involved in regulating metabolic processes related to obesity, influencing glucose and fat metabolism in humans and murine models [109,110].

*MRPL15* (mitochondrial ribosomal protein L15) is a nuclear gene that encodes a protein, which helps in protein synthesis within the mitochondrion. The *MRPL15* gene might be associated with carcass weight in pasture-finished beef cattle in Hawai’i [111] and in Korean Hanwoo cattle [112], milk fatty acids (C6:0) in Dual-Purpose Belgian Blue cows [113], residual feed intake in Australian Angus cattle [114], and body weight gain and feed intake in crossbred beef steers [115]. Given that the horses of this breed are kept on pastures year-round and face challenges related to food and forage availability during cold conditions, they have been under natural selection to regulate their energy for the long and cold winter season in Kazakhstan. Therefore, the *CMKLR1* and *MRPL15* genes most likely play crucial roles in adaptation of Mugalzhar horses to a year-round free-grazing system by mediating metabolic regulation and optimizing energy utilization to help sustain them through the winter when forage availability is restricted.

ZNF667 (zinc finger protein 667) facilitates DNA-binding transcription factor activity and sequence-specific binding to RNA polymerase II cis-regulatory regions. This gene, as a transcriptional regulator, helps to control the expression of resilience-related genes, which are critical for the cold climate conditions faced by the Mugalzhar breed in Kazakhstan. It has been reported to be under natural selection in the Bardigiano horse, a native Italian breed [116], the Rhenish German Draught Horse [117], Yorkshire pigs [118], and Tunchang pigs in China [119].

*CCDC66* (coiled-coil domain containing 66) encodes a microtubule-associated protein essential for ciliogenesis and cell division. It mediates protein transport to cilia, regulates spindle assembly during mitosis, and facilitates cytokinesis through microtubule organization. This gene has been reported to be associated with early-onset progressive retinal atrophy in Portuguese Water Dogs [120], generalized progressive retinal atrophy in Schapendoes dogs [121], and testicle length in chickens [122]. Since there is a lack of reports on the function of this gene in farm animals, including horses, further research is needed to investigate how it may aid adaptation in Mugalzhar horses. *LOC100055310* is not well annotated in the horse genome; however, it encodes a putative spermatogenesis-associated protein 31D3. This gene appears to be critical for reproduction and highly conserved, with two transcripts, 30 orthologues, and 25 paralogues.

### 4.4. OMIA Variants

Our findings showed that there is no deleterious variant segregating in this population. We identified seven variants, located within four genes, associated with Mendelian traits in horses that are segregating in this population. Three of the genes, including *MC1R*, *KIT*, and *MITF*, are associated with coat color in horses (OMIA 001199-9796, OMIA 000209-9796, and OMIA 000214-9796 [75]). Since coat color is not an economic trait in the Mugalzhar breed, the observed pattern is due to natural selection or most likely genetic drift during the breed formation. However, this breed is mainly dun or bay without spots.

Melanocortin 1 receptor (*MC1R*) is linked to the agouti-signaling-protein (*ASIP*) gene by a close epistasis relationship, such that relative amounts of melanin pigments in mammals are controlled by their antagonistic interaction [123]. The recessive missense mutation (ECA3:g.36979560C > T) of the *MC1R* gene determines the production of a red-yellow pigment (pheomelanin), while the other allele determines black pigment (eumelanin) [124]. If the dominant allele of *ASIP*, responsible for agouti signaling protein, is expressed, the melanocortin receptor 1 in the melanocytes is blocked, and consequently, pheomelanin synthesis happens. Hence, black, bay, and chestnut (three basic coat colors in horses) are results of the combination of specific genotypes of these two genes [124]. *KIT* (KIT proto-oncogene, receptor tyrosine kinase) regulates four depigmentation phenotypes: roan, sabino, tobiano, and dominant white. Haase et al. [125] reported that the dominant white color in different horse breeds is associated with multiple independent mutations inside this gene. However, this gene is crucial for the survival, proliferation, and differentiation of cells. Deficiency of KIT function causes lethal anemia, leading to prenatal or perinatal mortality [126].

*MITF* (melanocyte-inducing transcription factor) encodes a transcription factor containing basic helix-loop-helix and leucine zipper structural features. This transcription factor regulates pigment cell-specific expression of melanogenesis-related enzyme genes. The coat color pattern known as “splashed white” and defined by large white patterns on the legs, abdomen, and face, has been reported to be determined by this gene in several breeds of horse including American Paint [127,128], Quarter [129], Pura Raza Española horses [130], Thoroughbred [131], Menorca Purebred and Spanish Purebred [132] horses. Horses exhibiting this phenotype are typically deaf [127,129]. Since this breed relies on year-round grazing, the ability to evade predators (e.g., wolves) is critical for survival. This likely explains the significant decline in alleles associated with the phenotype over time.

A segregating stop-gain variant of the *DMRT3* (doublesex and mab-3 related transcription factor 3) gene was identified, which is associated with gaitedness. A mutation in this gene is mainly associated with gaitedness in horses, which can be found in horse breeds worldwide, not limited to a geographical area, as was found in 68 of the 141 horse breeds investigated by Promerová et al. [133]. The authors reported the allele frequency of this mutation in the studied breeds in a range of 1% to 100%, where it is more frequent in gaited and harness racing horse breeds [133]. This gene encodes an essential transcription factor that orchestrates vertebrate locomotion, functioning within spinal cord neural circuits to coordinate limb movement synchronization [134,135,136]. The Mugalzhar horse descended from the Jabe horse lineage, whose movement has been evolutionarily optimized for energy-efficient travel across vast steppes [137]. Consequently, the reduced heterozygosity observed in the studied breed likely resulted from strong natural selection pressures in their ancestral population.

Overall, genomic analysis of Mugalzhar horses at the whole genome sequence level revealed selection signatures within their genome that indicate a high level of adaptability to harsh weather conditions for a year-round grazing system.

## 5. Conclusions

This study provides the first comprehensive whole-genome analysis of the Mugalzhar horse breed, uncovering valuable insights into its genetic diversity, selection history, and adaptation mechanisms. Despite the limited sample size and geographic distribution, over 21 million high-quality variants were detected in this population. The high-impact exonic variants in the homozygote state for the alternative allele are located in the genes associated with fitness, adaptation, and reproduction, suggesting ancient natural selection footprints for the year-round grazing in Kazakhstan climate conditions. While no deleterious Mendelian variants were found, seven low-frequency heterozygous variants associated with coat color and gaitedness were detected. Our findings not only contribute to the understanding of Mugalzhar horse genetics but also establish a reference point for future conservation and breeding strategies aimed at improving performance and resilience in native horse populations. From a practical point of view, the identified variants can be integrated into breeding programs to enhance climate adaptation while preserving diversity in this breed, and also to apply this genomic framework to conserve other vulnerable native horse breeds facing environmental challenges.

## Figures and Tables

**Table 1:** Reads counts, sequence quality control (QC), and mapping statistics for each sample.

Horse ID	Read Count	Read (Raw/Trim)	QC > 20, %	QC > 35, %	Alignment, %
1	421,952,515	150/140	98.63	90.94	99.69
2	420,140,194	150/140	98.67	90.78	99.77
3	389,171,234	150/140	99.01	93.05	99.81
4	562,962,552	150/140	98.82	92.10	99.74
5	266,970,766	150/140	99.11	93.09	99.80
6	466,017,248	150/140	99.00	92.65	99.79
7	232,870,671	150/140	99.25	94.84	99.86
8	502,411,868	150/140	98.89	92.63	99.80
9	362,727,174	150/140	99.26	94.58	99.83
10	429,967,714	150/140	98.95	93.05	99.78
11	363,588,160	150/140	98.64	90.78	99.78
12	400,995,480	150/140	98.96	92.99	99.82
13	337,511,396	150/140	98.57	90.67	99.78
14	335,204,785	150/140	98.90	92.65	99.80
15	347,165,492	150/140	98.95	92.70	99.79
16	525,540,100	150/140	98.77	91.61	99.75
17	216,143,288	150/140	98.87	92.82	99.75
18	417,983,452	150/140	99.13	93.70	99.82
19	339,261,443	150/140	98.87	92.59	99.72
20	271,018,576	150/140	99.13	93.60	99.86

**Table 2:** Distribution of variants over 32 horse chromosomes.

ECC ^1^	All Variants			Bi-Allelic Variants		
	Indels	SNPs	Total	Indels	SNPs	Total
1 (NC_009144.3 ^2^)	147,722	1,357,453	1,505,175	126,369	1,349,565	1,475,934
2 (NC_009145.3)	97,645	899,301	996,946	83,781	893,743	977,524
3 (NC_009146.3)	93,450	868,813	962,263	80,354	863,936	944,290
4 (NC_009147.3)	91,653	852,027	943,680	78,769	846,792	925,561
5 (NC_009148.3)	76,667	690,517	767,184	65,553	686,424	751,977
6 (NC_009149.3)	74,571	674,296	748,867	63,869	670,070	733,939
7 (NC_009150.3)	79,382	727,068	806,450	68,070	722,632	790,702
8 (NC_009151.3)	80,305	776,161	856,466	69,441	771,166	840,607
9 (NC_009152.3)	64,599	603,074	667,673	55,591	599,656	655,247
10 (NC_009153.3)	72,890	649,547	722,437	62,265	645,511	707,776
11 (NC_009154.3)	48,745	419,897	468,642	41,227	417,041	458,268
12 (NC_009155.3)	41,910	427,686	469,596	37,294	423,529	460,823
13 (NC_009156.3)	38,426	363,283	401,709	32,834	360,942	393,776
14 (NC_009157.3)	73,042	668,303	741,345	62,609	664,494	727,103
15 (NC_009158.3)	72,635	679,471	752,106	62,273	675,408	737,681
16 (NC_009159.3)	68,916	635,961	704,877	58,733	632,351	691,084
17 (NC_009160.3)	69,046	642,029	711,075	59,593	638,266	697,859
18 (NC_009161.3)	71,768	664,346	736,114	61,759	660,204	721,963
19 (NC_009162.3)	54,275	508,176	562,451	46,645	505,024	551,669
20 (NC_009163.3)	74,882	693,542	768,424	66,477	683,845	750,322
21 (NC_009164.3)	50,108	484,203	534,311	43,275	481,038	524,313
22 (NC_009165.3)	39,885	385,028	424,913	34,402	382,654	417,056
23 (NC_009166.3)	43,983	398,492	442,475	37,631	396,114	433,745
24 (NC_009167.3)	40,132	376,887	417,019	34,381	374,407	408,788
25 (NC_009168.3)	30,733	291,433	322,166	26,275	289,800	316,075
26 (NC_009169.3)	38,294	381,680	419,974	33,366	379,107	412,473
27 (NC_009170.3)	36,748	348,404	385,152	31,760	346,037	377,797
28 (NC_009171.3)	36,445	347,184	383,629	31,480	345,095	376,575
29 (NC_009172.3)	32,311	313,570	345,881	28,037	311,406	339,443
30 (NC_009173.3)	28,951	269,626	298,577	25,025	267,744	292,769
31 (NC_009174.3)	22,581	212,724	235,305	19,245	211,489	230,734
X (NC_009175.3)	97,952	754,114	852,066	84,521	749,848	834,369
Total	1,990,652	18,364,296	20,354,948	1,712,904	18,245,338	19,958,242

^1^ ECC, *Equus caballus* chromosome; ^2^ NC_0091XX.X, NCBI Reference Sequence No. [61].

**Table 3:** Distribution of identified bi-allelic variants over functional annotation classes.

Functional Ontology Class	SNPs	Indels	Total
intergenic variant	13,745,973	1,243,963	14,989,936
intron variant	3,359,772	351,967	3,711,739
upstream gene variant	557,181	60,416	617,597
downstream gene variant	229,356	24,047	253,403
5′ UTR variant	132,691	14,854	147,545
3′ UTR variant	71,784	8073	79,857
missense variant	62,111	0	62,111
synonymous variant	42,319	0	42,319
non coding transcript exon variant	23,580	1289	24,869
splice region variant	13,331	2009	15,340
frameshift variant	0	4444	4444
splice donor variant	2610	323	2933
stop gained	2336	64	2400
start lost	988	49	1037
splice acceptor variant	669	156	825
inframe deletion	0	801	801
stop lost	509	12	521
inframe insertion	0	403	403
stop retained variant	126	7	133
protein altering variant	0	22	22
coding sequence variant	2	5	7
Total	18,245,338	1,712,904	19,958,242

**Table 4:** Descriptive statistics of inbreeding coefficients calculated using three different methods.

Inbreeding Estimation Method ^1^	Minimum	Maximum	Average
*F*_GRM_	−0.120	0.062	−0.038
*F*_HOM_	−0.149	0.188	−0.033
*F*_UNI_	−0.057	0.058	−0.033

^1^ *F*_GRM_, inbreeding coefficient driven from genomic relationship matrix (GRM); *F*_HOM_, Wright’s inbreeding coefficient based on the proportion of the loci with higher observed homozygosity than expected homozygosity; *F*_UNI_, Wright’s inbreeding coefficient based on the correlation between alleles in uniting gametes.

**Table 5:** Prioritized candidate genes harboring high-impact homozygous exon variants fixed in the Mugalzhar horse.

Gene Symbol	Ensembl Gene ID	No. of Variants	ECA ^1^	Start	End	No. of Orthologues	No. of Paralogues
*SCAPER*	ENSECAG00000017272	9	1	117,976,410	118,465,952	207	1
*FHAD1*	ENSECAG00000025126	8	2	37,672,050	37,824,768	142	1
*MMP15*	ENSECAG00000000196	6	3	10,831,201	10,851,185	273	22
*ADGRE1*	ENSECAG00000017237	5	7	4,879,448	4,948,792	103	50
*CMKLR1*	ENSECAG00000049382	10	8	14,730,554	14,789,710	344	7
*MRPL15*	ENSECAG00000012110	15	9	30,176,710	30,221,285	225	–
*ZNF667*	ENSECAG00000010995	6	10	25,714,426	25,740,647	175	7
*CCDC66*	ENSECAG00000018662	8	16	33,029,040	33,134,439	179	–
*LOC100055310*	ENSECAG00000035870	6	23	6,312,930	6,557,200	30	25

^1^ ECA, *Equus caballus* autosome.

**Table 6:** Variants associated with Mendelian traits and segregating in the Mugalzhar horse population.

Variants ^1^	A1 ^2^	A2 ^2^	MAF ^3^	No. Heterozygotes	Type of Variant	Gene	Phenotype
ECA3:g.36979560C > T	T	C	0.100	4	missense	*MC1R*	coat color, chestnut
ECA3:g.79538738C > T	T	C	0.025	1	missense	*KIT*	white spotting
ECA3:g.79548220T > C	T	C	0.025	1	missense	*KIT*	coat color, dominant white
ECA3:g.79566881T > C	C	T	0.025	1	missense	*KIT*	increased white spotting
ECA16:g.21555811delinsAAAT	A	C	0.025	1	deletion	*MITF*	splashed white
ECA16:g.21608936C > T	C	A	0.075	3	regulatory	*MITF*	white splashing
ECA23:g.22391254C > A	A	C	0.025	1	stop-gain	*DMRT3*	gaitedness

^1^ ECA: *Equus caballus* autosomes; ^2^ A1 and A2, alleles 1 and 2; ^3^ MAF, minor allele frequency.

## Data Availability

The raw fastq reads for all horses analyzed in the current study are available in the European Nucleotide Archive (ENA) under the study number PRJEB82323 (https://www.ebi.ac.uk/ena/browser/view/PRJEB82323 (accessed on 5 July 2025)). The detected SNPs and indels are available for download at the European Variants Archive (EVA) (https://www.omicsdi.org/dataset/eva/PRJEB82323 (accessed on 5 July 2025)).

## Supplementary Materials

The following supporting information can be downloaded at: https://www.mdpi.com/article/10.3390/ani15182667/s1, Supplementary Information: Mugalzhar horse history, breeding, production characteristics and sampling, Table S1: Horse gender, birth year and coat color for the collected samples, Table S2: The quality and quantity metrics for the extracted genomic DNA samples, Table S3: Summary of trimmed read statistics pooled for each horse sampled, Table S4: Distribution of the variants in different classes based on number of alleles.
