# Supplementary material for: Uncovering Genetic Diversity and Adaptive Candidate Genes in the Mugalzhar Horse Breed Using Whole-Genome Sequencing Data
Source: Animals (Basel). 2025 Sep 11;15(18):2667. doi: 10.3390/ani15182667 (PMC12466398; doi:10.3390/ani15182667)
Supplement: Supplementary file 1 [file animals-15-02667-s001.zip › Supplementary Information.pdf]

Supplementary Information: Mugalzhar Horse History, Breeding, Production Characteristics and Sampling

S1. Detailed Historical Background, Breeding Practices, and Production Characteristics

S1.1. History of the Breed

S1.1.1. Breed Creation

- The Mugalzhar horse breed was developed in Kazakhstan through the improvement of the native Kazakh horses of the Dzhabe type using reproductive crossing of local mares with Dzhabe stallions. The breed creation process was led by teams of Kazakhstani breeders and scientists, rather than a single individual, over several decades. The breed is notable for being the first meat-and-diary breed produced without improvement mating with stud farm breeds, relying, instead, on selection within a local gene pool. The development and scientific support for the breed have been provided by such institutions, among others, as the Kazakh Research Institute of Horse Breeding and Forage Production that continues to play a central role in research and genetic improvement of local breeds, including the Mugalzhar [1,2,3,4].

- Initial leading creators of the breed: Drs. Yu.N. Barmintsev, S.S. Rzabaev, I.N. Nechaev, N.A. Kikebaev, A.E. Zhumagul, and K.D. Bakhtybaev.

- After that, scientific research on testing of the Mugalzhar breed horses and improvement of their breeding and performance has been conducted by Drs. S.S. Rzabaev, T.S. Rzabaev, (I.N. Nechaev), (A.E. Zhumagul), N.A. Kikebaev, A.T. Turabaev, and G.T. Baktybaev.

S1.1.2. Breed Creation Timeline

The creation of the Mugalzhar breed spanned a timeline from 1969 to 1998, with the breed being officially recognized and approved as a distinct meat-and-diary horse breed in 1998. The registration and official recognition were carried out by the relevant state organs of the Republic of Kazakhstan, specifically under the Ministry of Agriculture that oversees all breed registration and livestock improvement programs [5].

S1.2. Current Population and Major Breeding Centers

The breed is structured into three main intra-breed types: Embinsky, Kulandinsky, and Kozhamberdinsky (the latter recognized since 2009), along with six lines and 55 families. The main breeding centers and farms for the Mugalzhar breed are located in the Aktobe, Kyzylorda, and Karaganda regions [3, 6]. In 2024, Iskhan et al. [2024] provided a description of a new stud farm type of the Mugaldzhar horse breed. It was named the Irtysh type and was developed in the village of Zhetizhar (Beskaragai District, Abai Region). The new type is characterized by high meat productivity (up to 58% meat yield), a high foaling rate (over 85%), and good adaptation to both hot climates and harsh winters [7].

S1.3. Main Production Enterprises

The primary production enterprises for the Mugalzhar breed are private farms and agricultural enterprises specializing in meat and milk production. Private farms account for the majority of horse meat production in Kazakhstan, with the Mugalzhar breed being a key contributor due to its high slaughter yield (55–60%) and significant milk production (up to 2,000 liters per lactation per mare). The breed is also used to improve the productivity of local herds in various regions, from the Priaral deserts to the high mountains of Eastern Kazakhstan [8,9].

S1.4. Year-Round Management and Environmental Adaptation

Mugalzhar horses are managed under extensive, year-round pasture conditions, primarily in the steppes, semi-deserts, and deserts of Kazakhstan. The breed is highly adapted to the harsh continental climate, withstanding extreme temperatures ranging from −40 °C in winter to 45 °C in summer. Horses often travel long distances (up to 30 km per day) to access water and forage, and are capable of accumulating fat reserves to survive periods of undernutrition. Their robust constitution, dense winter coat, and efficient metabolism enable them to thrive on coarse, sparse vegetation and endure the environmental challenges of the Kazakh steppe. These adaptive traits are the result of both natural and artificial selection over centuries, making the Mugalzhar breed exceptionally suited for year-round grazing and low-input management systems [8,9].

S2. Detailed Information on the Sampling Site and the Studied Samples

S2.1. Geographic Location Name and Coordinates of the Sampling Site

City of Khromtau, Shalkar District, Aktobe Region, Western Kazakhstan.
Geographical coordinates of Khromtau, Kazakhstan:

- Latitude: 50°15′04″ N
- Longitude: 58°26′24″ E

Elevation above sea level: 429 m.

S2.2. Owner of Sampled Animals: Breeding Center and Production Enterprise

Amandyk Zhumagalyuly, Head, ORDA breeding farmer enterprise (https://www.instagram.com/kh_orda_04/ (accessed on 4 July 2025)).
All 20 sampled individuals belong to the Aktobe population of the Mugalzhar horse breed.

S2.3. Characterization of Sampled Animals

S2.3.1. General Origin of Sampled Individuals

All 20 sampled animals belong to the Kozhamberdinsky intra-breed type. This type (formerly known as Sary-Arkinsky) was previously one of the three main intra-breed types of the Mugaldzhar breed [Iskhan et al., 2019]. Within the Kozhamberdinsky intra-breed type, the Sary-Arkinsky stud farm (factory) subtype is currently recognized as well as the Meiman line that was the only source of all 20 samples.

S2.3.2. Description of the Sary-Arkinsky Factory Subtype

The Sary-Arkinsky factory subtype is part of the structure of the Mugaldzhar breed as one of the two factory types alongside the Kaindinsky subtype. This subtype is part of the selective breeding work aimed at improving the breed and developing its specialized directions.

S2.3.3. Description of the Meiman Line

Within the Kozhamberdinsky intra-breed type of the Mugaldzhar breed, the Meiman line was established. This line represents a selective breeding achievement developed within this intra-breed type.

S2.3.4. Age and Sex of the Sampled Animals

The sampled individuals were 5–6 years old, except one outstanding breeding stallion that was 19 years old and has a nickname of Parasat. In all, the sampled group included nine stallions and 11 mares (see for more details the Supplementary Information Table).

Supplementary Information Table. Inventory list of the Mugalzhar breed horses from the Aktobe population (Amandyk Zhumagalyuly, Head, ORDA breeding farm enterprise, Khromtau, Shalkar District, Aktobe Region, Kazakhstan).

No.	Nickname, gender and age group	Coat color	Year of birth, identification number, line	Sampled biological material	Photo
1	Parasat, breeding stallion	buckskin	2005	blood	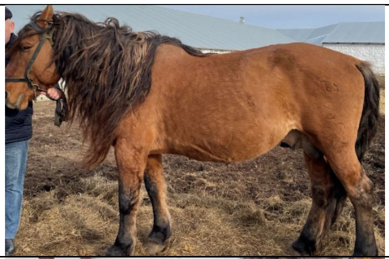
2	Zubay, producer stallion	bay	2019,<br>line: outstanding stallion Altyn Zhal	blood	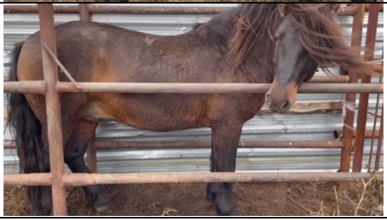

3 (4)	Bolek, sire stallion	bay	2019-1, line: outstanding stallion Borsyk	blood	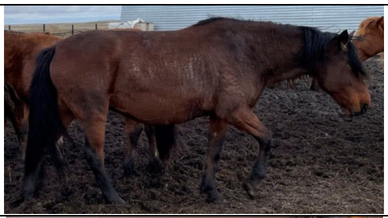
4 (6)	Kyzyl Zhal, sire stallion	chestnut	2021-9, line: outstanding stallion Python	blood	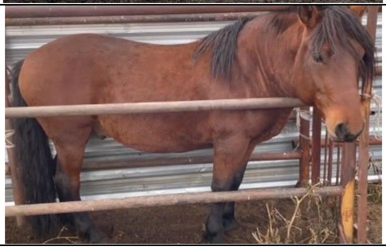
5 (7)	Mai Kara, sire stallion, champion of the breed 2022	brown (dark bay)	2017, 35-17, line: outstanding stallion Maupas	blood	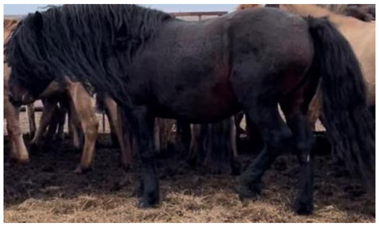
6 (8)	mare	buckskin	2017, line: outstanding stallion Koktas	blood	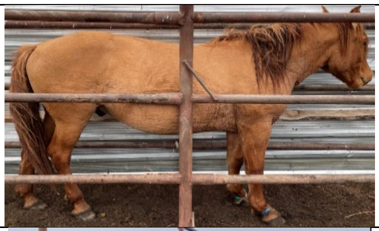
7 (9)	mare	buckskin	2021, 2-04, line: outstanding stallion Zaman, champion of the Exhibition of Achievements of National Economy (VDNKh)	blood	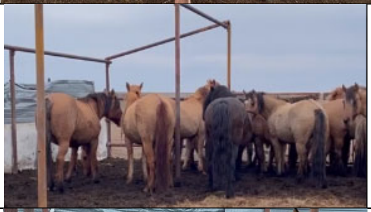
8 (10)	Baktory, sire stallion	bay	2019, 423-19, line: outstanding stallion Cormorant, son of Baggeldy	blood	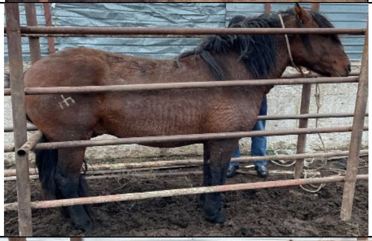
9 (11)	mare	chestnut (white spot on forehead)	2019 , MK-19	blood	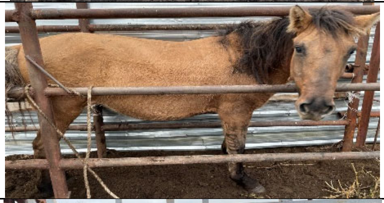
10 (12)	stallion	chestnut	2020, 555-20	blood	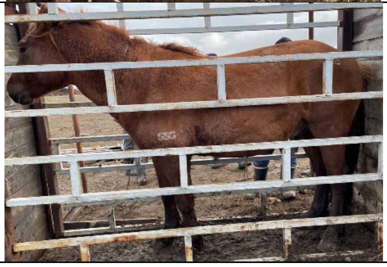

11 (13)	mare	brown (dark bay)	MK 19-282	blood	-
12 (14)	mare	bay	MK 19-283	blood	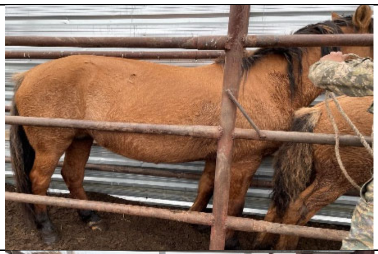
13 (15)	mare	flaxen (game)	MK 19	blood	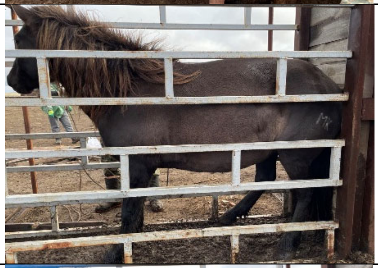
14 (16)	mare	dark bay	MK 19-80	blood	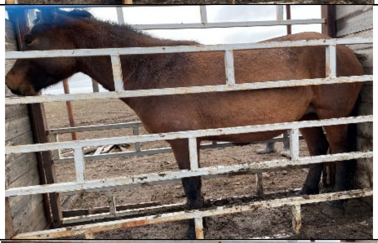
15 (17)	mare	bay (kerauyz)	MK 19-100	blood	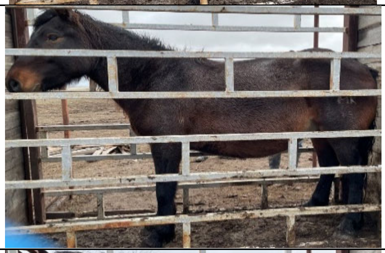
16 (18)	Batu, stallion	bay	11-18, line: Borsyk	blood	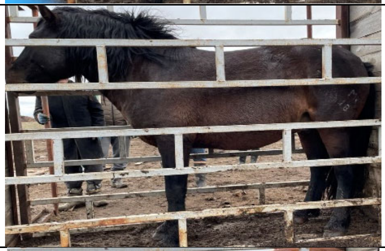
17 (19)	mare	brown (white spot on forehead)	MK 19-78	blood	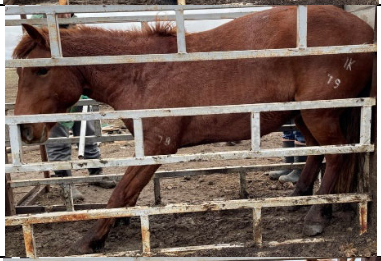
18 (21)	mare	chestnut	2017, MZ	blood	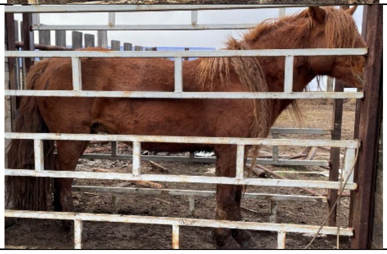

19 (22)	Maidan, stallion	bay	MK 2018, line: 85	blood	–
20 (23)	mare	bay	MK 19-78	blood	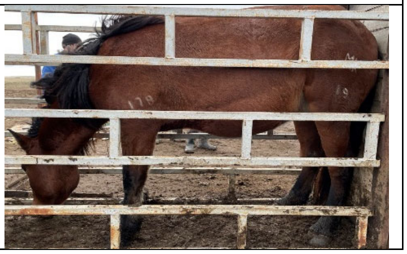

References

1. Rzabayev, S.S. [*Mugalzhar Horse Breed*]; LLP Information and Printing Center—Kokzhiyek: Aktobe, Kazakhstan, 2007.
2. Satybaldin, A.A. Current State of Horse Breeding and Horse Sports in Kazakhstan. In Proceedings of the First International Conference; n.p.: Kostanay, Kazakhstan, 2002. Available online: https://scholar.google.com/scholar?q=Current+State+of+Horse+Breeding+and+Horse+Sports+in+Kazakhstan (accessed on 26 June 2025).
3. Orazymbetova, Z.; Ualiyeva, D.; Dossybayev, K.; Torekhanov, A.; Sydykov, D.; Mussayeva, A.; Baktybayev, G. Genetic diversity of Kazakhstani *Equus caballus* (Linnaeus, 1758) horse breeds inferred from microsatellite markers. *Vet. Sci.* **2023b**, *10*, 598. https://doi.org/10.3390/vetsci10100598
4. Kabylbekova, D.; Assanbayev, T.S.; Kassymbekova, S.; Kantanen, J. Genetic studies and breed diversity of Kazakh native horses: a comprehensive review. *Adv. Life Sci.* **2024**, *11*, 18–27. Available online: https://www.als-journal.com/1113-24/ (accessed on 26 March 2024).
5. Pozharskiy, A.; Abdrakhmanova, A.; Beishova, I.; Shamshidin, A.; Nametov, A.; Ulyanova, T.; Bekova, G.; Kikebayev, N.; Kovalchuk, A.; Ulyanov, V. Genetic structure and genome-wide association study of the traditional Kazakh horses. *animal* **2023**, *17*, 100926. https://doi.org/10.1016/j.animal.2023.100926
6. Seleuova, L.A.; Naimanov, D.K.; Jaworski, Z.; Aubakirov, M.Z.H.; Mustafin, M.K.; Mustafin, B.M.; Safronova, O.S.; Baktybaev, G.T.; Turabaev, A.T.; Domatski, V.N. Population genetic characteristic of horses of Mugalzhar breed by STR-markers. *Biomed. Res.* **2018**, *29*, 3508–3511. https://doi.org/10.4066/biomedicalresearch.29-18-1041
7. Iskhan, K.; Uskenov, R.; Akimbekov, A.; Baymukanov, D.; Yuldashbayev, Y.; Orynaliev, K. The Irtysh factory type of the Mugalzhar breed and the line Zamana, Bakay. *Ìzdenìster, nəťiželer* [*Res. Results*] **2024**, *4*(104), 16–24. https://doi.org/10.37884/4-2024/02
8. Iskhan, K.Z.; Kalashnikov, V.V.; Akimbekov, A.R.; Mongush, S.D.; Demin, V.A.; Rzabayev, T.S.; Nesipbaeva, A.K.; Zhilkybaeva, M.M.; Zhikishev, Y.K. Zootechnic characteristics of modern populations of Mugalzhar horse breed. *Bull. Natl. Acad. Sci. Rep. Kazakhstan* **2019**, *6*(382), 75–82. https://doi.org/10.32014/2019.2518-1467.147
9. Shamshidin, A.S.; Beishova, I.S.; Alikhanov, O.; Aubakirov, K.A.; Shamekova, M.K.; Kargaeva, M.T.; Karibayeva, D.K.; Baimukanov, D.A. [Productive longevity of Mugalzhar mares]. *Ġylym žəňe bìlìm* [*Sci. Educ.*] **2025**, *2*, 279–287. https://doi.org/10.52578/2305-9397-2025-1-2-279-287
