# Supplementary material for: Uncovering Genetic Diversity and Adaptive Candidate Genes in the Mugalzhar Horse Breed Using Whole-Genome Sequencing Data
Source: Animals (Basel). 2025 Sep 11;15(18):2667. doi: 10.3390/ani15182667 (PMC12466398; doi:10.3390/ani15182667)
Supplement: Supplementary file 1 [file animals-15-02667-s001.zip › Supplementary Tables S1-S4.pdf]

Supplementary Table S1. Horse gender, birth year and coat color for the collected samples.

Horse ID	Gender	Birth year	Coat color
1	stallion	2005	buckskin
2	stallion	2019	bay
3	stallion	2019	bay
4	stallion	2021	chestnut
5	stallion	2017	brown
6	mare	2017	buckskin
7	mare	2021	buckskin
8	stallion	2019	bay
9	mare	2019	chestnut
10	stallion	2020	chestnut
11	mare	2019	brown
12	mare	2019	bay
13	mare	2019	flaxen
14	mare	2019	dark bay
15	mare	2019	bay
16	stallion	2018	bay
17	mare	2019	brown (white spot on forehead)
18	mare	2017	chestnut
19	stallion	2018	bay
20	mare	2019	bay

Supplementary Table S2. The quality and quantity metrics for the extracted genomic DNA samples.

Sample No.	Concentration, ng/µl	A260 absorbance	A280 absorbance	A260/A280 ratio	A260/A230 ratio
1	269.0	5.380	2.890	1.86	2.22
2	363.0	7.259	3.903	1.86	2.23
3	375.4	7.508	4.037	1.86	2.26
4	331.9	6.638	3.570	1.86	2.21
5	315.8	6.316	3.398	1.86	2.21
6	379.6	7.592	4.070	1.87	2.22
7	389.8	7.797	4.146	1.88	2.39
8	325.7	6.513	3.497	1.86	2.23
9	465.7	9.314	5.003	1.86	2.25
10	243.2	4.864	2.633	1.85	2.17
11	366.6	7.333	3.933	1.86	2.25
12	469.6	9.392	5.054	1.86	2.26
13	237.9	4.758	2.569	1.85	2.14
14	215.2	4.305	2.323	1.85	2.15
15	274.5	5.489	2.962	1.85	2.15
16	278.5	5.570	3.009	1.85	2.24
17	316.9	6.337	3.413	1.86	2.23
18	292.5	5.849	3.146	1.86	2.21
19	304.7	6.093	3.277	1.86	2.22
20	220.3	4.406	2.376	1.85	2.16

Supplementary Table S3. Summary of trimmed read statistics pooled for each horse sampled.

Horse ID	Total sequences, mln	%GC	%Deduplicated
1	422	43.000	87.24346
2	420	43.000	87.68652
3	389	43.000	88.90265
4	563	43.000	87.83946
5	267	43.000	88.20028
6	466	42.875	88.22897
7	233	42.500	87.06770
8	502	43.100	88.13093
9	363	42.500	86.15962
10	430	40.375	89.24361
11	364	43.000	86.31979
12	401	43.000	88.70293
13	338	43.000	86.90059
14	335	43.000	88.08527
15	347	43.000	88.23448
16	526	43.000	87.21769
17	216	43.000	87.36865
18	418	43.000	87.36886
19	339	43.000	87.09315
20	271	43.500	87.60197

Supplementary Table S4. Distribution of the variants in different classes based on number of alleles.

Number of alleles	SNPs	Indel	Total
2	18,245,338	1,712,904	19,958,242
3	110,872	213,921	324,793
4	6,675	49,324	55,999
5	1,130	11,405	12,535
6	228	2,601	2,829
7	53	497	550
Total	18,364,296	1,990,652	20,354,948
